# Pyruvate kinase M2 fuels multiple aspects of cancer cells: from cellular metabolism, transcriptional regulation to extracellular signaling

**DOI:** 10.1186/s12943-018-0791-3

**Published:** 2018-02-19

**Authors:** Ming-Chuan Hsu, Wen-Chun Hung

**Affiliations:** 1 National Institute of Cancer Research, National Health Research Institutes, No. 367, Shengli Road, Tainan, 704 Taiwan; 2 Institute of Medicine, College of Medicine, Kaohsiung Medical University, Kaohsiung, 802 Taiwan

## Abstract

Originally identified as a metabolic enzyme that catalyzes the transfer of a phosphate group from phosphoenolpyruvate (PEP) to ADP in the glycolytic pathway, pyruvate kinase M2-type (PKM2) has been shown to exhibit novel biological activities in the nucleus and outside the cells. Although cell-based studies reveal new non-canonical functions of PKM2 in gene transcription, epigenetic modulation and cell cycle progression, the importance of these non-canonical functions in PKM2-mediated tumorigenesis is still under debate because studies in genetically modified mice do not consistently echo the findings observed in cultured cancer cells. In addition to regulation of gene expression, the existence of PKM2 in exosomes opens a new venue to study the potential role of this glycolytic enzyme in cell-cell communication and extracellular signal initiation. In this review, we briefly summarize current understanding of PKM2 in metabolic switch and gene regulation. We will then emphasize recent progress of PKM2 in extracellular signaling and tumor microenvironment reprogramming. Finally, the discrepancy of some PKM2’s functions in vitro and in vivo, and the application of PKM2 in cancer detection and treatment will be discussed.

## Background

Biochemical analysis by charactering the enzymatic activity that catalyzes the formation of lactate from glucose in cell lysates revealed the first intracellular metabolic pathway, the glycolytic pathway. Beginning from the purification of fractions that contained glycolytic activity, a number of pioneer researchers contributed to the identification of enzymes that involve in each step in the pathway [1–3]. These results build up our modern concept in the interchange of aerobic and anaerobic respiration and energy production under various physiological and pathological circumstances.

The existence of an enzyme that catalyzed the production of ATP by transferring a phosphate group from PEP to ADP in the liver was first reported in 1934 [4]. Subsequent isolation of the enzyme, known as pyruvate kinase (PK) later, demonstrated differences in tissue distribution and catalytic kinetics suggesting this enzyme may have different isoforms [5–8]. During 1982 to 1984, various PK genes were cloned from yeast, chicken and rat [9–12]. The functional study of PKM2 was initiated by the identification of a candidate gene in mouse in early 1980s [11]. Later, Noguchi et al. showed that two isoforms of PK (PKM1 and PKM2) are encoded by the same *PKM* gene via alternative splicing [12]. In human, PKM isoforms are also produced via a similar splicing mechanism by including exon 9 and 10 into *PKM1* and *PKM2* mRNA separately [13].

Several findings caught researcher’s attention to the potential role of PKM2 in tumorigenesis. First, PKM2 is the embryonic isoform that highly expressed during animal development. Its transcription is attenuated in a number of adult tissues while it is reactivated in tumors [14, 15]. Second, study of the relative abundance of PKM1 and PKM2 in normal and tumor tissues demonstrated a switch from the PKM1 isoform to the PKM2 isoform in various cancers like hepatocellular carcinoma [16, 17]. Third, the change of mRNA splicing from *PKM1* to *PKM2* is enhanced by c-Myc oncogene suggesting cancer cells actively engage in this switch to fit their requirement in proliferation and metabolism [18]. Fourth, modulation of PKM2 activity by activators or inhibitors affect tumor growth in vivo [19–21].

### The first episode: PKM2 as a metabolic enzyme in the cytoplasm

**Fig. 1:**
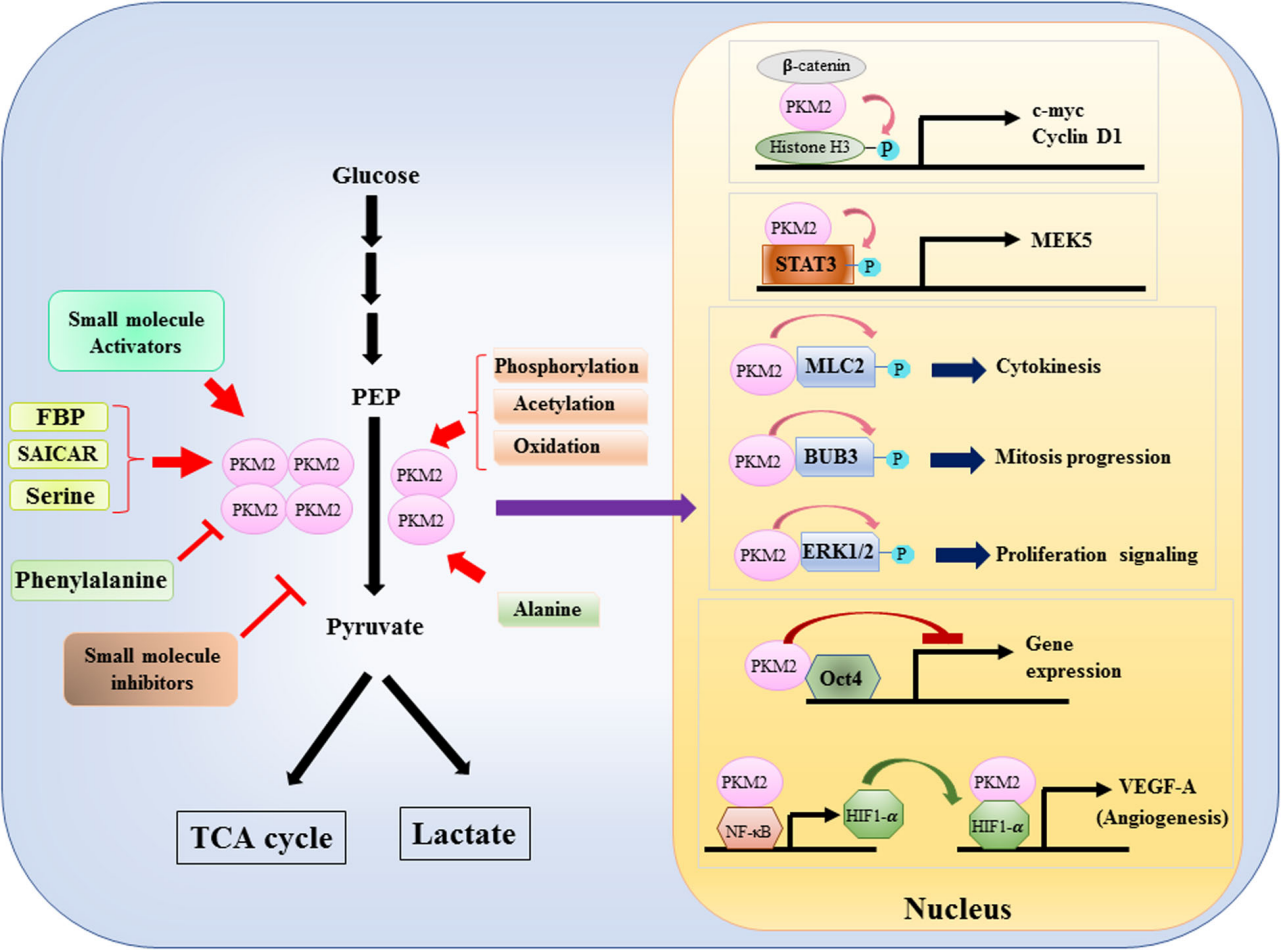
Modulation of PKM2 activity by physiological molecules and post-translational modification and the potential role of PKM2 in transcriptional regulation

Since the role of PKM2 in metabolic control of glycolysis in cancer cells has been extensively reviewed [22–24], we only summarize three crucial differences between PKM1- and PKM2-mediated catalysis and cellular metabolism here. The first difference is subunit interaction. Both PKM1 and PKM2 are tetrameric proteins formed by four identical subunits. Each subunit (or monomer) contains four structural domains including A, B, C, and N-terminal domain. The monomer first dimerizes together and then two dimers interact via the dimer-dimer interface orchestrated by the C domain of monomer to form a tetramer. Because PKM1 and PKM2 include different exons in their mRNAs, this changes the encoded amino acids in the C domain and alters the tetramer stability. Under physiological condition, PKM1 constitutively organizes as a tetramer while PKM2 can be existed in tetramer or dimer. The second difference is allosteric regulation. Depending on the intracellular concentrations of small molecules and metabolites, the activity of PKM1 and PKM2 can be differentially regulated. One of the most well-known allosteric regulators is fructose-1,6-bisphosphate (FBP). This glycolytic intermediate directly binds PKM2 and increases the affinity of PKM2 for PEP [25]. On the contrary, FBP does not significantly affect PKM1 activity. In addition to FBP, other metabolites, amino acid and small molecules have been reported to affect PKM2 activity [19, 26–31] (Fig. 1). However, the concentration required for activation or inhibition is high and the modulatory effect is modest. Whether these small molecules play an important role in the control of PKM activity under physiological circumstances is still unclear. PKM2 activity is also regulated by post-translational modification, such as phosphorylation, acetylation and oxidation, which favor the low activity of dimeric PKM2 (Fig. 1). The third difference is energy production and intermediate utilization. Since PKM1 constitutively exists as the active tetramer, the main biological function of this isoform is the generation of ATP to supply cellular energy. However, PKM2, in addition to produce ATP, can switch to the less active dimeric form to generate several glycolytic intermediates which can be used as building blocks for the biosynthesis of amino acids, lipids and nucleotides.

### The second episode: PKM2 as a signaling modulator in the cytoplasm

In addition to function as a glycolytic enzyme, PKM2 is proposed to involve in more cellular processes due to the identification of interacting proteins in the cytoplasm. For example, PKM2 was shown to be an interacting protein of several tyrosine kinases including A-Raf, Break point cluster region-Abelson (BCR-ABL) fusion kinase, fibroblast growth factor receptor 1 (FGFR1) etc. [32, 33]. These binding partners have been shown to modulate the dimeric/tetrameric change of PKM2 to alter cell metabolism. However, it is possible that PKM2 may reciprocally affect the catalytic kinetics, substrate binding and cytoplasmic location of these binding partners to modulate signal transduction. The finding that PKM2 is a phosphor-tyrosine binding protein strengthens this possibility because many intracellular signaling mediators can bind to phosphor-tyrosine residue to assemble specific protein complexes for signal transmission [34]. To date, the list of PKM2 binding partners grows continuously. We highlight several new members and discuss their biological implication here. Mukheriee et al. demonstrated that PKM2 could bind with HuR, a RNA binding protein which plays an important role in the control of mRNA stability and translational efficiency, to promote cell cycle progression and proliferation of glioma cells [35]. Interestingly, another RNA binding protein tristetraprolin which could bind a number of mRNA via the AU-rich element at 3′-untranslational region (3’-UTR) was also found to be a PKM2 interacting partner, and PKM2 induced phosphorylation and degradation of tristetraprolin to modulate breast cancer growth [36]. These two studies imply a potential translational control function of PKM2. Recently, Liang et al. identified the anti-apoptotic protein Bcl2 as a new PKM2 partner [37]. They demonstrated that oxidative stress induced the translocation of PKM2 into mitochondria where it phosphorylated and stabilized Bcl2 by preventing its degradation via ubiquitination-dependent pathway. These data suggested that PKM2 helps cancer cells to adapt oxidative stress elicited by intracellular metabolic change or extracellular insult.

### The third episode: PKM2 as a transcriptional regulator in the nucleus

A nuclear role of PKM2 in the regulation of gene transcription or epigenetic modification was firstly suggested by the finding that PKM2 bound with Y333-phosphorlated β-catenin, and the β-catenin-PKM2 complex was recruited to the nucleosomes to phosphorylate histone H3 at threonine 11 [38] (Fig. 1). This phosphorylation subsequently increased histone H3 acetylation that led to upregulation of β-catenin target genes. Another transcription factor directly phosphorylated by PKM2 is signal transducer and activator of transcription 3 (STAT3) [39]. PKM2-mediated phosphorylation of STAT3 at tyrosine 705 enhanced STAT3 activity to upregulate the expression of mitogen-activated protein kinase kinase 5 (MEK5). Beside transcription factors, PKM2 has been shown to phosphorylate myosin light chain 2 (MLC2), BUB3 and extracellular signal-regulated kinase 1 and 2 (ERK1 and ERK2) [40–42]. Interestingly, PKM2 also acts via phosphorylation-independent manner to affect gene expression. For example, PKM2 has been found to bind with Oct4, one of the master transcription factors that control self-renewal of stem cells, and inhibit Oct4-mediated transcription [43]. PKM2 can also enhance tumor angiogenesis by interacting with NF-κB and HIF-1α in the nucleus and activating the expression of HIF-1α target gene VEGF-A. Consequently, increased secretion of VEGF-A boosts blood vessel formation which contributes to tumor growth [44]. Although these studies strongly suggested the nuclear localization and protein kinase function of PKM2 in various physiological and pathological circumstances, however the importance of nuclear PKM2-mediated gene expression has been challenged by studies using PKM2 knockout cells. By using [^32^P]-labeled PEP and PKM2-null mouse embryonic fibroblasts, Hosios et al. showed that PEP-dependent phosphorylation is not a common event in cells and the reaction is not catalyzed by PKM2 [45]. The discrepancy of these studies is currently unresolved and the protein kinase activity of PKM2 needs further confirmation.

### The fourth episode: PKM2 as an extracellular signaling communicator

The presence of extracellular PKM2 opens a new avenue for the study of PKM2 biological function. Buschow et al. provided the first evidence that PKM2 could be detected in B-cell exosomes and was identified as a MHC class II-associated protein [46]. Two subsequent studies also indicated that PKM2 is existed in exosomes released by various cells [47, 48]. Currently, several public databases like ExoCarta and EVpedia provide comprehensive information for the components including proteins, lipids, nucleic acids of extracellular vesicles in different species. All of the data confirm that PKM2 is a package protein of exosomes. Recent studies have clearly demonstrated a communicative role of exosomes by delivering different components from host cells to recipient cells [49–51]. It is expectable that PKM2 may play a role in cell-cell crosstalk.

Emerging evidence indeed support this hypothesis. For example, a recent study demonstrated that blood circulating PKM2 may promote tumor growth and angiogenesis by increasing the growth, migration and matrix adhesion of endothelial cells [52]. Another investigation also showed that PKM2 secreted from colon cancer cells might act via an autocrine stimulation to enhance cell migration by activating the PI3K/Akt and Wnt/β-catenin pathways [53]. In addition to cancer cells, neutrophils at the tissues damage sites could release PKM2 to promote angiogenesis and wound healing [54]. Our recent study also demonstrated that recombinant PKM2 protein could induce phosphorylation and activation of epidermal growth factor receptor (EGFR) [55]. Moreover, we found that R339E mutant PKM2 which preferentially formed dimeric PKM2 activated EGFR more significantly than the tetrameric PKM2. Keller et al. identified 154 proteins as potential substrates for PKM2 after treatment of Hela cells with succinyl-5-aminoimidazole-4-carboxamide-1-ribose-5′-phosphate (SAICAR), an intracellular metabolite which could stimulate the protein kinase activity of PKM2 [42]. They also found EGFR as a PKM2 substrate. Their results are different from ours in two ways. First, the signaling pathways activated in our study are elicited by extracellular PKM2 while the molecular targets identified in their study are potential substrates of intracellular PKM2. Second, increase of ERK1/2 activity in our study is initiated by EGFR activation while ERK1/2 activation in their study is directly stimulated by the SAICAR/PKM2 complex. One similar phenomenon observed in both studies is that R339E mutant PKM2 activates signaling molecules more significantly than the wild type PKM2 suggesting the distinct role of dimeric and tetrameric PKM2 in oncogenesis. By using receptor tyrosine kinase array, we found that extracellular PKM2 only activated limited growth factor receptors in breast cancer cells (data not shown). Currently, the selectivity of receptor activation by extracellular PKM2 remains unknown. In addition, why R339E mutant PKM2 is more potent in the activation of EGFR is also not clear. More experiments are needed to answer these questions.

**Fig. 2:**
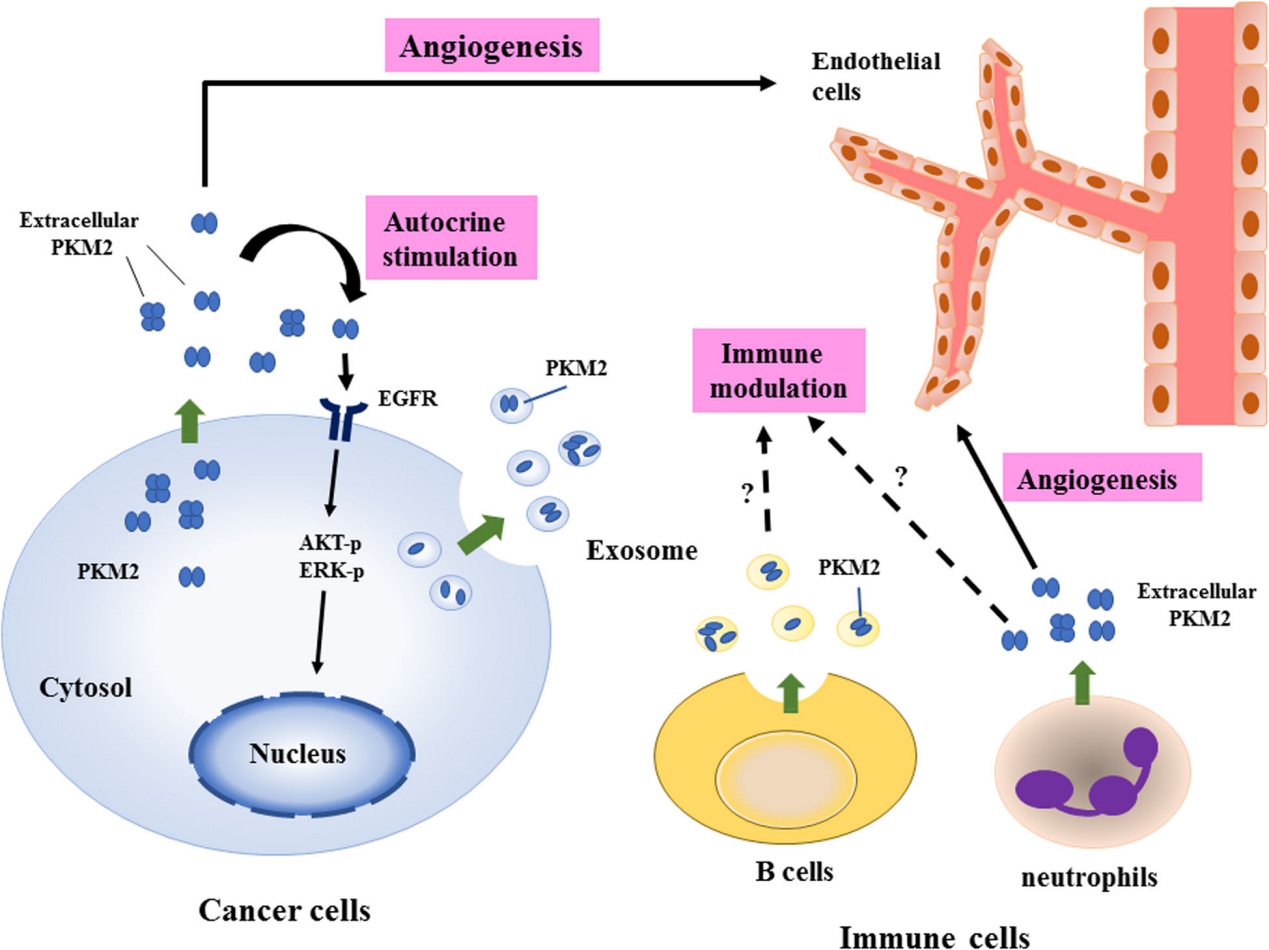
The potential tumor-promoting effect of extracellular PKM2

Another elegant question to be addressed is whether free PKM2 and vesicle-packaging PKM2 exhibit similar effect in promoting tumorigenesis (Fig. 2). Free extracellular PKM2 could not penetrate plasma membrane and could only activate intracellular signaling via cell surface proteins like growth factor receptors. Conversely, vesicle-packaging PKM2 could be endocytosed by cancer and stroma cells, and the PKM2 released from vesicles could affect metabolism and gene expression via intracellular mechanism. The PKM2-null cells or mice will be useful to elucidate whether these two extracellular forms of PKM2 could act synergistically or antagonistically in tumorigenesis.

### The fifth episode: unanswered discrepancy of PKM2

In addition to the cell-based data discussed above, the oncogenic role of PKM2 has also been challenged after the generation of PKM2 knockout mice. Israelsen et al. generated a conditional knockout mouse model by deleting the PKM2-specific exon 10 [56]. Surprisingly, depletion of PKM2 accelerated but not attenuated tumor formation driven by loss of *Brca1* gene in mice. These data indicated that PKM2 is not required for the proliferation of cancer cells. Interestingly, PKM1 expression was only detected in non-proliferating tumor cells suggesting a tumor-suppressive role of PKM1 in breast cancer. In addition, PKM2 knockout mice have a high incidence to develop hepatocellular carcinoma spontaneously after a long latency due to the imbalance in metabolism [57]. These results against the notion that PKM2 plays an oncogenic role in vivo.

### The continuing episode: is PKM2 a cancer biomarker and drug target?

Although the results of genetically engineering mouse model do not support the tumor-promoting activity of PKM2, overexpression of PKM2 is universally found in human cancers and is associated with poor clinical outcome (Table 1) [58–81]. Two recent meta-analysis studies also supported this conclusion. Wu et al. analyzed the data of 2812 patients with solid tumors of digestive system obtained from 16 cohort studies and found that overexpression of PKM2 is associated with reduced overall survival in gastric cancer, esophageal squamous cell carcinoma, hepatocellular carcinoma, biliary cancer and oral cancer [82]. However, PKM2 is not a prognostic factor for pancreatic cancer. This finding is not consistent with previous studies showing that increase of PKM2 is an indicator of poor survival of pancreatic cancer patients [71, 73, 77]. Another investigation included 4796 cases from 27 individual studies demonstrated that PKM2 upregulation is correlated with worse overall survival, disease-free survival and recurrence-free survival in pooled data [83]. However, stratified by cancer type, PKM2 does not predict a poor survival of pancreatic cancer. Collectively, PKM2 seems to be a liable prognostic marker in most of solid tumors.

**Table 1:** Prognostic significance of PKM2 in human cancers

Cancer	Detection	Gene	Expression	Prognosis	Ref.
Mesothelioma	qRT-PCR	PKM2, ARHGDIA	Increased	Poor	[58]
		TM4SF1, COBLL1			
Chordoma	IHC	PKM2, ENO1, gp96	increased	poor	[59]
Gastric cancer	q-RT-PCR/IHC	PKM2	increased	poor	[60]
Gallbladder cancer	IHC	PKM2, ACVR 1C	increased	poor	[61]
		GAPDHS, TYRP1			
Melanoma	IHC	PKM2, GAPDH	increased	poor	[62]
Ovarian cancer	q-RT-PCR/IHC	PKM2, GAPDH, ATP5B	increased	poor	[63]
Tongue cancer	IHC	PKM2, LDH5	increased	poor	[64]
Esophageal cancer	IHC/WB	PKM2, HK1, PFKB	increased	poor	[65]
Hepatoma	IHC	PKM2	increased	poor	[66]
Hepatoma	IHC	PKM2, TRIM35	increased	poor	[67]
Hepatoma	IHC	PKM2, Bim	increased	poor	[68]
Cervical cancer	IHC	PKM2	increased	poor	[69]
Oral cancer	IHC	PKM2	increased	poor	[70]
Pancreatic cancer	IHC	PKM2, HK2	increased	poor	[71]
Breast cancer	IHC	PKM2, VEGF-C	increased	poor	[72]
Pancreatic cancer	IHC	PKM2	increased	poor	[73]
Gastric cancer	IHC	PKM2, HK1	increased	poor	[74]
Cholangiocarcinoma	IHC	PKM2	increased	poor	[75]
Colorectal cancer	IHC	PKM2	increased	poor	[76]
Pancreatic cancer	IHC	PKM2, LDHA	increased	poor	[77]
Gallbladder cancer	IHC	PKM2	increased	poor	[78]
Osteosarcoma	IHC	PKM2	increased	poor	[79]
Gastric cancer	IHC	PKM2	increased	poor	[80]
Ovarian cancer	IHC	PKM2	increased	poor	[81]

*Abbreviation*: *q-RT-PCR* quantitative reverse-transcriptase polymerase chain reaction, *ARHGDI* Rho GDP dissociation inhibitor alpha, *TM4SF1* transmembrane 4 L six family member 1, *COBLL1* Cordon-Bleu WH2 repeat protein like 1, *ENO1* enolase-1, *gp96* heat shock protein 90 beta family member 1, *ACVR 1C* activin A receptor type 1C, *GAPDH* glyceraldehyde-3-phosphate dehydrogenase, *GAPDHS* glyceraldehyde-3-phosphate dehydrogenase, spermatogenic, *TYRP1* tyrosinase related protein 1, *ATP5B* mitochondrial ATP synthase beta subunit, *LDH5* lactate dehydrogenase 5, *HK1* hexokinase 1, *PFKB* phosphofructokinase-2, *TRIM35* Tripartite motif containing 35, *HK2* hexokinase 2, *VEGF-C* vascular endothelial growth factor C

On the contrary, the use of PKM2 as a diagnostic factor is controversial. A proteomic analysis demonstrated that PKM2 is a potential diagnostic marker for the detection of lung cancer [84]. However, a recent study suggested PKM2 is not a good diagnostic marker for lung cancer due to low specificity [85]. Similarly, PKM2 alone is unlikely to be a useful marker for the screening of colon cancer [86]. However, combination of multiple markers could increase sensitivity and specificity for cancer diagnosis [86].

The therapeutic potential of PKM2 is an intriguing event in cancer treatment. From one side, inhibition of PKM2 is expected to inhibit glycolysis, impair gene transcription and suppress cellular proliferation. Therefore, PKM2 inhibitors seem to be good candidates for anti-cancer drug development. By using library screening, Vander Heiden et al. identified three novel classes of PKM2 inhibitors and showed that the most effective compound inhibited PKM2 activity and induced death of cancer cells [31]. Recently, Ning et al. found that novel naphthoquinone derivatives are potent PKM2 inhibitors [87]. One effective compound 3 k suppressed the proliferation of multiple cancer cell lines at sub-micromolar concentrations while it showed little detrimental effect on normal cells. From the other side, activation of PKM2 may also inhibit tumor growth. Because the low activity PKM2 dimer is the major isoform that triggers glycolysis in the cytoplasm and gene transcription in the nucleus in cancer cells, PKM2 activators which can promote the formation of tetrameric PKM2 may switch glycolysis to mitochondria pathway and reduce nuclear entry to attenuate gene transcription. Both effects impair metabolic demand and growth-supporting signaling that leads to tumor regression. Two pioneer studies identified various PKM2 activators and characterized their specificity in vitro [88, 89]. A subsequent study demonstrated that PKM2 activators indeed promoted tetramer formation and suppressed tumor growth in vivo [19]. These results suggested PKM2 activators could be promising anti-cancer drugs.

**Fig. 3:**
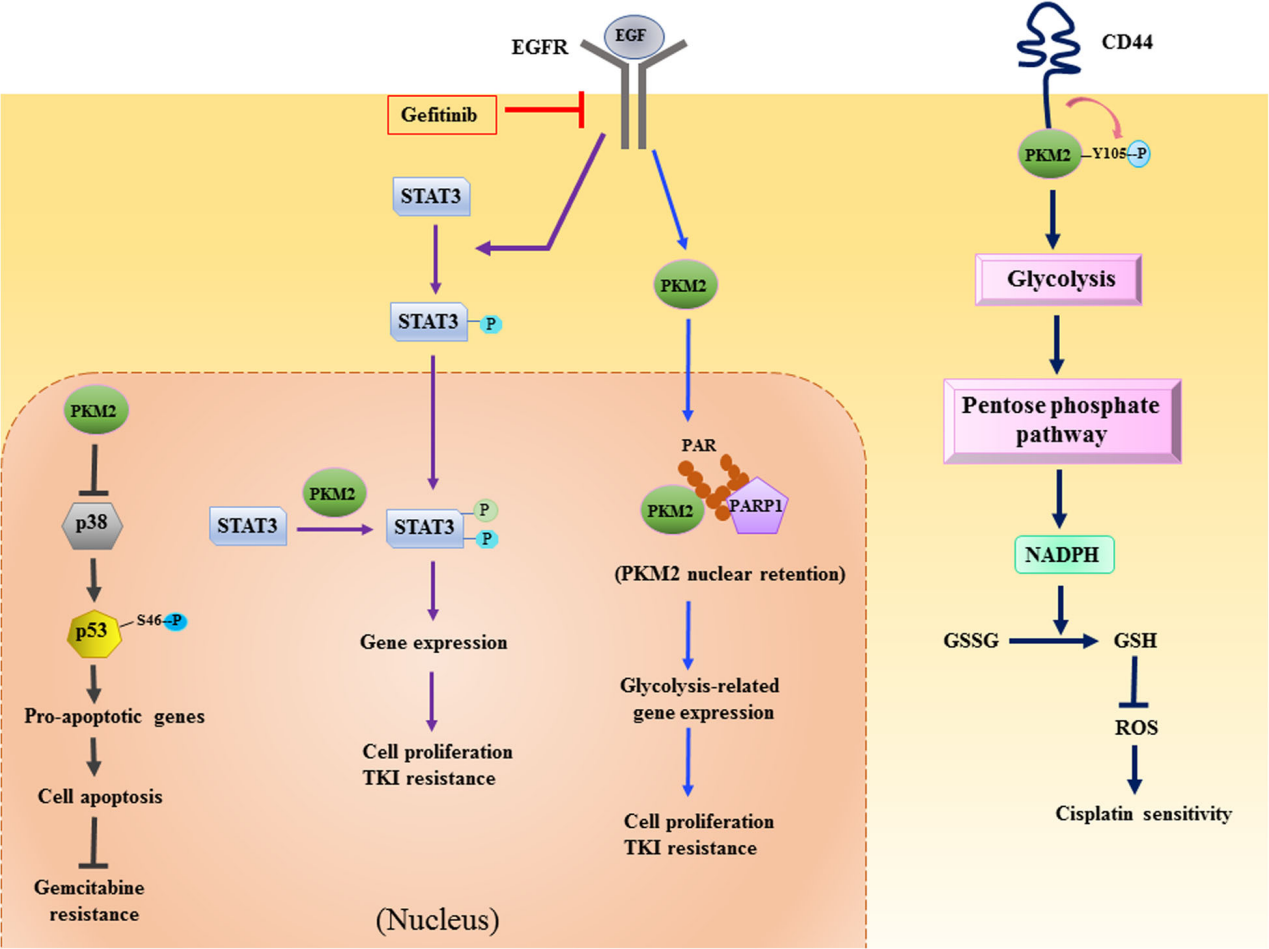
PKM2 contributes to the proliferation and drug resistance in cancer cells

Resistance to chemotherapy is a major blockage for cancer treatment. Overcoming the inherent chemoresistance of cancer cells is urgent for clinical research. The roles of PKM2 in chemoresistance of cancer cells have been revealed, and targeting PKM2 has been shown to re-sensitize chemoresistant cancer cells. A recent study showed that CD44 interacts with PKM2 and suppresses PKM2 activity via increasing Tyr105 phosphorylation of PKM2. CD44 ablation induced the switch from aerobic glycolysis to mitochondrial respiration and increasing reactive oxygen species (ROS) production, resulting in the enhancement of cisplatin sensitivity in colorectal cancer cells [90]. Inhibition of PKM2 activity was demonstrated to suppress glycolysis and overcome cisplatin resistance. Therefore, the combination of cisplatin and PKM2 inhibitors may be an effective strategy for chemotherapy (Fig. 3). PKM2 has also been reported to participate in the regulation of gemcitabine resistance in pancreatic cancer cells. Kim et al. showed that PKM2 promotes gemcitabine resistance through negatively regulating p38-mediated p53 phosphorylation, therefore reducing transcriptional activity of p53 and suppressing the expression of pro-apoptotic genes [91]. Moreover, ectopic expression of R399E-PKM2, which preferentially forms dimeric PKM2, enhances the resistance of pancreatic cancer cells to gemcitabine. These evidences suggest PKM2 contributes to the induction of drug resistance via a non-metabolic mechanism. Because dimeric PKM2 acts mainly as a transcriptional regulator in cancer cells, PKM2 activators which promote the formation of tetrameric PKM2 may be effective in suppressing non-metabolic function of PKM2 and reducing resistance to gemcitabine in pancreatic cancer cells. Nuclear PKM2 has also been demonstrated to contribute to the resistance of EGFR inhibitor in colorectal cancer and lung cancer [92, 93]. Li et al. showed that nuclear PKM2-mediated STAT3 phosphorylation reduces the sensitivity of colorectal cancer cells to gefitinib and disruption of the interaction of nuclear PKM2 and STAT3 restored gefitinib sensitivity in the cells [92]. More recently, another study demonstrated that PKM2 translocates into the nucleus and interacts with poly-ADP ribose (PAR) upon growth factor stimulation. The PAR-binding activity of PKM2 is critical for nuclear retention and gene transcription of PKM2 and is important for the promotion of cell proliferation and tumor growth. They concluded that inhibition of PKM2 nuclear function may overcome the resistance of EGFR-mutated cancer cells [93]. Both studies pointed out a crucial role of nuclear PKM2 in mediating drug resistance in cancers and suggested targeting nuclear PKM2 may be a promising strategy to override the resistance.

## Conclusions

Our understanding of PKM2 function expands dramatically in the past two decades. Originally identified as a metabolic enzyme in glycolysis, PKM2 is now found to be a multi-face protein that fuels different aspects of cancer cells to sustain tumor growth. The use of PKM2 as a prognostic marker has been validated in a variety of cancers. In addition, the application of PKM2 activators or inhibitors in cancer therapy can be expected in the coming decade.
